# Anthocyanins Extracted from *Oryza sativa* L. Prevent Fluorouracil-Induced Nuclear Factor-κB Activation in Oral Mucositis: In Vitro and In Vivo Studies

**DOI:** 10.3390/ijms19102981

**Published:** 2018-09-29

**Authors:** Salunya Tancharoen, Prana Shakya, Somphong Narkpinit, Pornpen Dararat, Kiyoshi Kikuchi

**Affiliations:** 1 Department of Pharmacology, Faculty of Dentistry, Mahidol University, Bangkok 10400, Thailand; pornpen.dar@mahidol.ac.th (P.D.); kikuchi_kiyoshi@kurume-u.ac.jp (K.K.); 2 Maxillofacial Prosthetic Service, Department of Prosthodontics, Faculty of Dentistry, Mahidol University, Bangkok 10400, Thailand; pranashakya@gmail.com; 3 Department of Pathobiology, Faculty of Science, Mahidol University, Bangkok 10400, Thailand; somphong.nar@mahidol.ac.th; 4 Division of Brain Science, Department of Physiology, Kurume University School of Medicine, Kurume 8300011, Japan

**Keywords:** anthocyanin, NF-κB, 5-fluorouracil, chemotherapy, oral mucositis

## Abstract

This study aims to investigate the immunomodulatory effect of anthocyanins (ANTs) from *Oryza sativa* L. extracts on 5-fluorouracil (5-FU)-induced oral mucositis, using a rat model and oral keratinocytes. ANTs were detected by high-performance liquid chromatography (HPLC)-electrospray ionization mass spectrometry. Animals were randomly given varying doses of ANT-rich extract treatment (500 mg/kg and 1000 mg/kg) in the absence or presence of 5-FU-induced mucositis. Buccal mucosae were photographed and scored for macroscopic analysis and incisional biopsies of cheek pouches were collected for microscopic examination of oral mucositis. 5-FU caused marked hemorrhage, extensive ulcerations and abscesses compared to non-treated animals with slight erythema. Histologically, a loss of collagen bundles and inflammatory cell infiltrates was observed. After 29 days of ANT treatment, lesions resolved, and abundant collagen fibers were evident in the lamina propria. Buccal mucosa of 5-FU-injected rats showed increased Nuclear factor-kappa B (NF-κB) p50 and p65 in oral keratinocytes. The administration of ANT reduced NF-κB-positive cells in 5-FU rats (*p* < 0.001) compared to the non-treatment group. In oral keratinocytes, ANT treatment significantly restored 5-FU-induced growth inhibition and impaired the nuclear accumulation of NF-κB p50 and p65. Our study demonstrated that ANT from *Oryza sativa* L. exhibited effective anti-inflammatory properties against 5-FU-induced oral mucositis by inhibiting NF-κB activation.

## 1. Introduction

Oral mucositis is a significant complication of chemotherapy and/or radiation therapy, occurring in 80–100% of patients undergoing “high-risk” regimens and 40% of patients receiving standard dose chemotherapy [1]. Initially, oral mucositis manifests as erythematous lesions 4–5 days following chemotherapy, progresses to erosive and ulcerative lesions within 7–10 days and persists for 2–4 weeks [2]. Lesions associated with mucositis result in severe pain, difficulty in speech and swallowing, and a decreased quality of life for the patient. Furthermore, it predisposes the patient to a higher risk of local and systemic infection and interferes with the ability to deliver the intended dose of cancer therapy, thereby posing a threat to patient life, as well as causing an increased length of hospital stay for the patient [3,4]. 5-fluorouracil (5-FU) is a common chemotherapeutic agent which is widely used in the treatment of solid tumors [5,6]. 5-FU is an antimetabolite drug that acts as a phase-specific anti-pyrimidine by inhibiting DNA synthesis via inhibition of the enzyme thymidylate synthase [7,8]. Additional molecular mechanisms underlying the anticancer activities of 5-FU have been reported, such as via the inhibition of the mammalian target of rapamycin (mTOR)/ribosomal protein S6 kinase pathway activation [9]. 5-FU was also found to inhibit cancer cell migration by upregulating Sestrin-2 [10].

Systemic chemotherapy with 5-FU causes toxicity to the entire gastrointestinal tract. These cytotoxic effects are particularly evident in the oral and the oropharyngeal mucosa due to its rapid rate of epithelial cell turnover. Oral mucositis is not only a consequence of direct injury to the oral basal cells, but is also caused by a series of complex biological events based on five overlapping biological phases: initiation, message generation, signal amplification, ulceration, and healing [11]. The mechanism of 5-FU-induced oral mucositis has been well identified. Likewise, the activation and translocation of transcription factor, nuclear factor-κB (NF-κB), from the cytosol to the nucleus is one of the key elements in the initiation of 5-FU-induced mucositis that leads to the upregulation of genes and the subsequent production of pro-inflammatory cytokines such as tumor necrosis factor-α (TNF-α), interleukin (IL)-1β, and IL-6 [2,11], cyclooxygenase (COX)-2 [12], and high-mobility group box 1 protein (HMGB1) [13].

The consumption of anthocyanins (ANTs) has a wide range of health-promoting properties [14]. ANTs are members of the flavonoid group of phytochemicals that have been shown to have antioxidative properties and potential in therapeutic immunomodulation, leading to anti-cancer [15,16] and anti-inflammatory effects [17,18]. In vitro, ANTs from black soybean seed coats prevent excessive inflammation by blocking the translocation of NF-κB p65 into the nucleus in human dermal fibroblasts and keratinocytes [17]. In an in vivo rat model of acute lung inflammation, ANT from blackberries was shown to reduce inflammatory parameters in a dose-dependent manner [19]. Similarly, human models have revealed that ANT could suppress NF-κB activation in monocytes and reduce the plasma concentration of pro-inflammatory mediators [18]. Recently, Tao, et al. reported that the administration of ANT from red cabbage (*Brassica oleracea* L. var. *capitata* L.) could prevent irinotecan-induced intestinal mucositis [20]. Black rice (*Oryza sativa* L.) has been widely cultivated and consumed in Southeast Asian countries for a long time. ANT in black rice was able to restore liver enzyme activity in rats with liver damage through its antioxidant properties [21]. In addition, ANTs were also shown to inhibit breast cancer cell proliferation [22]. Despite these findings, there have been no investigations into the effects of black rice-derived ANT in oral mucositis.

Although a variety of new approaches to oral mucositis have been employed, an efficacious intervention for prophylaxis or management of oral mucositis has not yet been identified. Based on our ANT study, and the critical role of NF-κB activation in the initiation of oral mucositis, the aim of this study was to evaluate the protective effects of ANT-rich extract found in *Oryza sativa* L. on oral wound healing, using an experimental rat model of 5-FU-induced mucositis, as well as attempting to elucidate possible transcription factor mechanisms.

## 2. Results

### 2.1. Identification of Anthocyanins (ANT) in Oryza sativa L.

**Figure 1:**
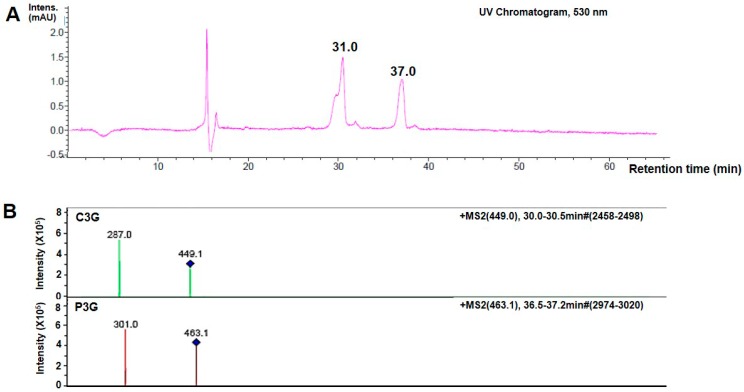
HPLC-electrospray ionization (ESI) mass spectrometry (MS) analysis of anthrocyanins (ANTs) in *Oryza sativa* L. (**A**) Representative HPLC chromatograms of Cyanidin-3-glucoside (C3G) and Pelargonidin-3-glucoside (P3G) in the extracts at 530 nm. Peaks were detected with a retention time at 31.0 and 37.0 min, identified as C3G and P3G, respectively; (**B**) Fragmentation patterns in the mass spectra of C3G and P3G in the ANT. The assay was performed in triplicate.

The ANT composition of *Oryza sativa* L. was determined by HPLC-electrospray ionization (ESI) mass spectrometry (MS) HPLC-ESI-MS. The ANT were purified using a Hypersil Gold C18 column and the resulting chromatogram at 530 nm is shown (Figure 1A). A chromatogram within the retention time of 30–30.5 min indicated the presence of Cyanidin-3-glucoside (C3G); in addition, a chromatogram within the retention time of 36.5–37.2 min, was identified as Pelargonidin-3-glucoside (P3G). Fragmentation patterns in the mass spectra of C3G and P3G in the ANT are shown in Figure 1B. Molecular ion (M+) at *m*/*z* 449.1, and a fragment ion at *m*/*z* 287.0 indicated the presence of C3G, whereas M+ at *m*/*z* 463.1 and a fragment ion at *m*/*z* 301, was identified as P3G. The parameters used for ANT peak identification is demonstrated in Table 1. Results of an ultraviolet (UV)-visible (Vis) wavelength spectra quantification of total ANT showed that the phenolic-rich extract contained ≈ 60 mg/g of total ANT (calculated as C3G equivalents). In the crude extracts, the total antioxidant capacity, ferric-reducing ability of plasma (FRAP) and total phenolic content of ANT-rich extracts were 1.07 ± 0.06 mM/g, 27.23 ± 1.00 mM Fe (II) equivalents/gram of fresh weight (gFW) and 181.73 ± 12.79 mM Gallic acid equivalent (GAE)/gFW, respectively (Table 2). Additionally, total antioxidant capacity and total phenolic content were significantly higher in the purified extracts compared to the crude extracts (*p* < 0.05), whereas there were no statistically significant differences (*p* > 0.05) in FRAP levels.

### 2.2. General Conditions and Body Weight of Subjects

**Figure 2:**
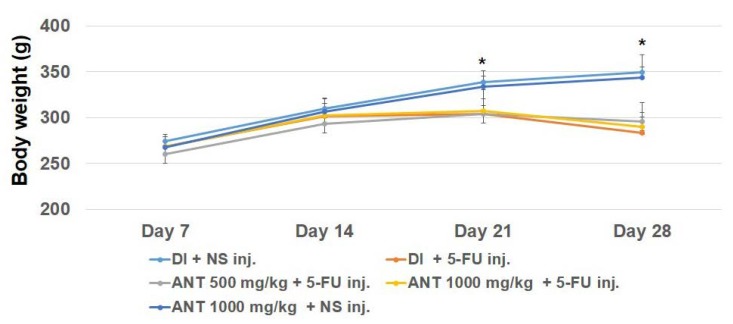
Mean body weight of rats in different groups. Rats were gavage-fed with either deionized water (DI) or ANT-rich extract and injected with normal saline (NS) or 60 mg/kg of 5-fluorouracil (5-FU). Body weight was measured every two days. Data expressed as mean ± S.D. (*n* = 6 rats/group), * *p* < 0.05, vs. rats in the 5-FU treatment groups.

Changes in body weight were observed in the five groups of rats for 29 days (Figure 2). Rats in the groups B, C and D which were subjected to 5-FU injection showed a significant progressive loss of body weight (*p* < 0.05) on day 21 (303.8 ± 16.6, 303.7 ± 29.6, and 307.6 ± 22.9) compared to their respective non-5-FU controls in the group A (333.8 ± 12.4) and E (333.8 ± 11.9). In the group which underwent 5-FU injection, the general condition of the animals, as reflected in their weight loss, became poor; however, the groups under pretreatment with ANT-rich extract were less morbid. During the experimental period, no mortality was seen in the rats that had received ANT-rich extract. Rats in group A and E showed no general health problems.

### 2.3. Oral Mucositis Lesions Are Attenuated in ANT-Pretreated Rats

**Figure 3:**
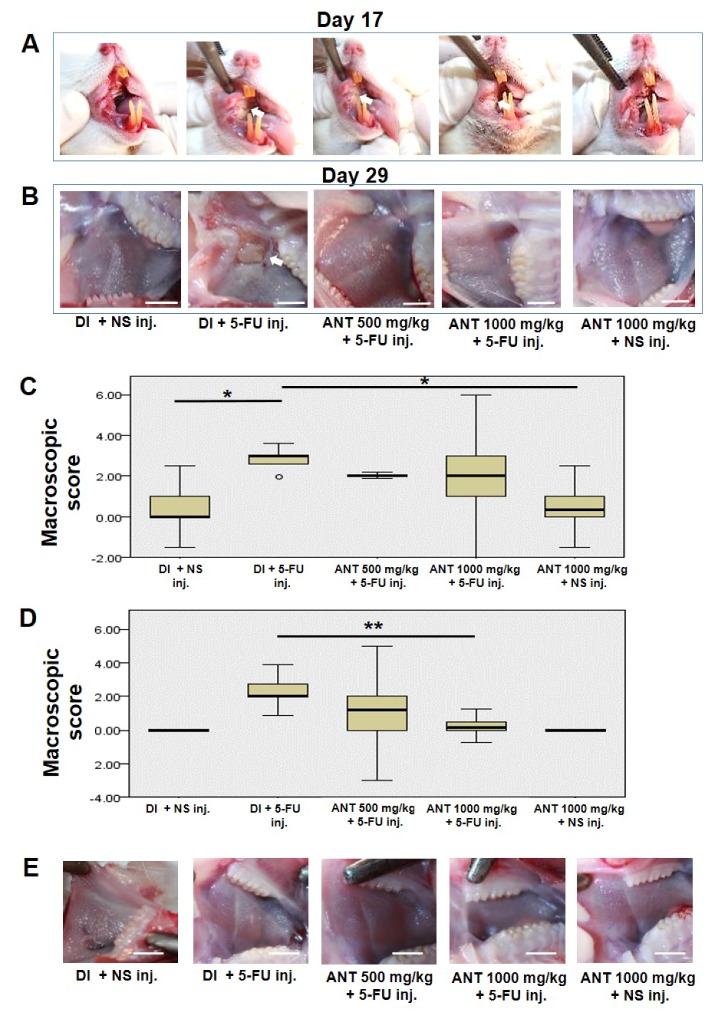
Pretreatment of rats with ANT-rich extract attenuates 5-FU-induced oral mucositis lesion. Rats were gavage-fed with either deionized water (DI) or ANT-rich extract and injected with normal saline (NS) or 5-FU. Macroscopic analysis of the oral mucositis lesion at the right buccal mucosa was observed on (**A**) day 17 and (**B**) day 29. Arrows indicate oral mucositis lesion. Scale bar, 10 mm; the box plots of the macroscopic scores of the mucositis lesion on (**C**) days 17 and (**D**) days 29 were measured by three examiners. Data are expressed as mean ± S.D., *n* = 6, Kruskall–Wallis test, * *p* < 0.001, ** *p* < 0.01. Box plots represent the median levels and the 25th and 75th percentiles of the observed data; whiskers represent the 5th and 95th percentiles in each group, and outliers (๐); (**E**) macroscopic analysis of the left buccal pouch without mechanical trauma on day 29. Scale bar, 10 mm.

On day 17, animals were randomly assigned with a high macroscopic analysis score of oral mucositis at the right side of their cheek pouch (Figure 3A). Severe clinical manifestations of oral mucositis patterns such as erythema, extensive ulcerations, and abscesses were shown in groups B, C and D which were subjected to 5-FU injection. On day 29, rats in group B showed a macroscopic pattern of accentuated erythema, extensive ulcers, and abscesses (Figure 3B). Interestingly, rats in group C showed generalized erythema without abscesses and hemorrhage. Animals receiving ANT-rich extract intake for 1000 mg/kg (group D) showed lesion healing without erythema. Figure 3C,D show the box plot of the macroscopic scores of the mucositis lesion on days 17 and 29, respectively. On day 17, the macroscopic scores of the lesion in group B were predominately grade 2 and 3 (2.83 ± 0.27), and the values were higher when compared to group C (2.05 ± 0.1) and group D (2.0 ± 0.9). In addition, the administration of deionized water (DI) (group A) or ANT-rich extract alone (group E) revealed a significantly lowered macroscopic score (*p* < 0.001) compared to group B. On day 29, a significant decrease of macroscopic score (*p* < 0.01) was found in group D (0.27 ± 0.4) but not group C (1.05 ± 0.9) compared to group B (2.28 ± 0.4). Furthermore, no mucositis lesions were observed at the left buccal mucosa of all rats throughout the experiment (Figure 3E). The macroscopic scores on days 17 and 29 were measured by three examiners. The Pearson’s correlation coefficient (r) was used to show the correlation between the examiners (Figure S1). According to the macroscopic results obtained, we concluded that pretreatment with ANT-rich extract 1000 mg/kg in 5-FU rats for 29 days exhibited a significant recovery of wound healing compared to rats receiving 5-FU alone.

### 2.4. Effect of ANT-Pretreatment on Histopathological Aspects of 5-FU-Induced Oral Mucositis

**Figure 4:**
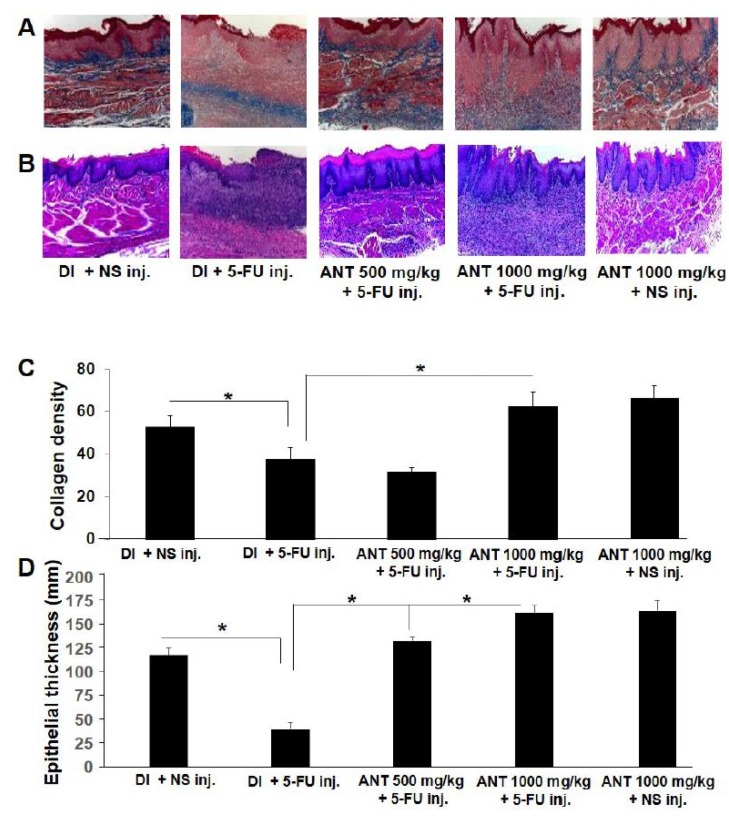
Histological examination of buccal mucosa in 5-FU induced oral mucositis. (**A**) Mallory’s azan staining; (**B**) hematoxylin and eosin staining. Note that blue indicates collagen bundles stained by Mallory’s azan; original magnification ×40; (**C**) collagen density and (**D**) epithelial thickness were quantitated and data are expressed as mean ± S.D. * *p* < 0.001.

Collagen is the primary component of the extracellular matrix in connective tissue [23,24]. Closely comparable histopathological results of oral mucositis lesions were obtained compared with the macroscopic findings. At day 29, Mallory’s azan-stained buccal mucosae in 5-FU-injected rats without ANT-rich extract pretreatment (group B) showed a severe loss of collagen bundles in the connective tissue (Figure 4A). For the ANT-rich extract pretreatment group (groups C and D), well-organized, parallel, densely packed and thick bundles of collagen fibers were demonstrated in the lesion, whereas collagen fibers were prominently mature in the oral mucosae of non-5-FU control rats (group E). In addition, a corresponding hematoxylin and eosin (H and E)-stained wound section is shown for granulation tissue and inflammatory cells in the lesion (Figure 4B). Examination of the non-5-FU-injected group without oral mucositis (groups A and E) revealed normal rat buccal mucosae with normal epithelium and connective tissue without inflammatory infiltration. Rats in group B showed granulation tissue with an abundant accumulation of mononuclear cells around the blood vessels, the presence of hemorrhagic areas, and active inflammation, but inflammation was attenuated in the ANT-rich extract-pretreatment group. Oral mucositis lesions with healing in group C revealed vascular ingurgitation, areas of re-epithelialization, inflammatory cell infiltration with neutrophil prevalence, and the absence of hemorrhagic areas, edema, ulceration or abscesses. These results were more pronounced in group D with no discrete erythema, no hemorrhagic areas or abscess, comparable to the histopathologic examination in group E. The amount of collagen was quantified in respective tissues (Figure 4C). The average collagen level in group B was significantly lower than that in the non-5-FU control (group A) (37.8 ± 5.4 and 52.8 ± 4.9; *p* < 0.001). The average collagen level in group D was significantly higher than in group B (62.5 ± 6.7 and 37.8 ± 5.4; *p* < 0.001). The oral epithelium thickness was also compared among the groups (Figure 4D). The average level of epithelial thickness in group B was significantly lower than that in group A (40.73 ± 6.4 and 118.8 ± 8.2; *p* < 0.001). In the groups which underwent ANT-rich extract treatment, the epithelial thickness levels were significantly higher than in group B (*p* < 0.001). These data indicate that ANT-rich extract at a level of 1000 mg/kg can significantly improve wound healing parameters, including epithelialization and collagen formation, in 5-FU-induced oral mucositis.

### 2.5. ANT Suppressed 5-FU-Induced Transcription Factor NF-κB p50 and p65 Activation in Oral Mucositis

**Figure 5:**
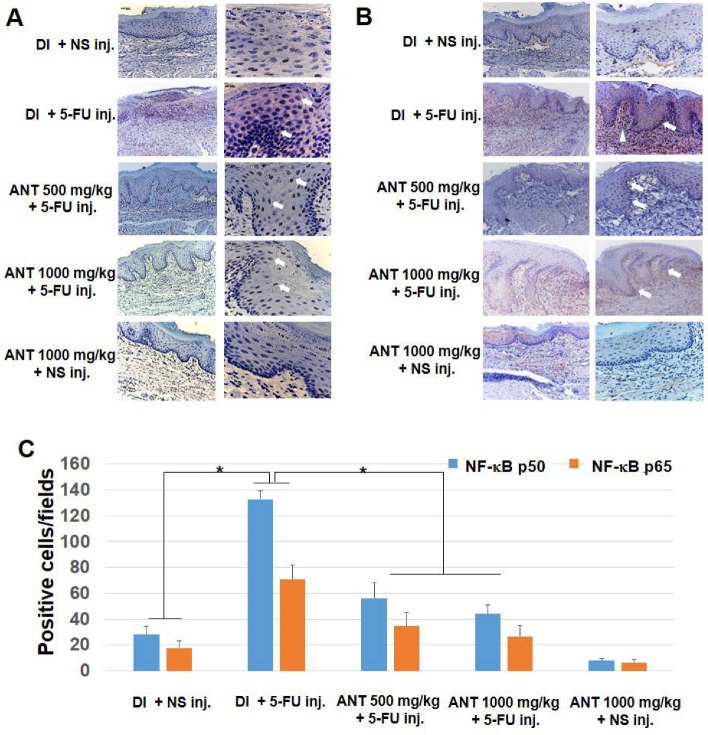
Localization of NF-κB p50 and p65 isoform in oral mucositis. By immunohistochemistry, slides were stained with (**A**) anti-NF-κB p50 Ab; (**B**) anti-NF-κB p65 Ab. Nuclei were counterstained with Mayer’s hematoxylin. Arrows indicate positive stained nuclei in oral epithelial cells. The arrowhead indicates inflammatory cells. Original magnification ×10 (left images) and ×40 (right images); (**C**) the number of NF-κB-positive cells per field. Data are expressed as the mean ± S.D. Error bars indicate standard deviation (*n* = 6), * *p* < 0.001.

To identify the mechanism underlying ANT-rich extract treatment in oral mucositis pathology, we assessed the presence of NF-κB protein nuclear translocation in the tissues by immunohistochemical staining. Our findings demonstrated the nuclear localization of NF-κB p50 and p65 within the stratum granulosum and spinosum in the oral epithelium, and inflammatory cells in the granulation tissues of all rats injected with 5-FU, although the number of positive cells varied between groups (Figure 5A). Larger amounts of nuclear staining with anti-NF-κB p50 Ab and anti-NF-κB p65 Ab (Figure 5B) were demonstrated in the epithelial cells and inflammatory cell infiltrates in the sample of rats in group B than in group A. Positive nuclear staining for NF-κB p50 and NF-κB p65 within the aggregates were less intense in the sample of rats pretreated with ANT-rich extract for 500 mg/kg (group C) and 1000 mg/kg (group D) in a dose-dependent manner compared to rats in group B. The quantification of positive immunostaining cells shows that abundant amounts of activated NF-κB p50 and p65 were significantly observed throughout the oral keratinocytes of the rats in group B compared to group A (*p* < 0.001) (Figure 5C). Similarly, after 29 days of administration with ANT-rich extract, a significant reduction of the positive staining of NF-κB in groups C and D was observed compared to group B (*p* < 0.001). Moreover, no positive staining of the immunoglobulin G (IgG) isotype control was observed in all tissues (Figure S2).

### 2.6. Pretreatment with ANT for 1000 mg/kg Blocks HMGB1 Levels in 5-FU-Induced Oral Mucositis

**Figure 6:**
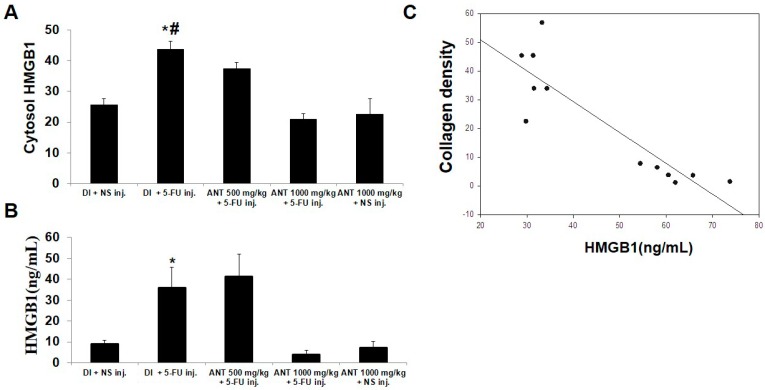
The Evaluation of cytosolic and serum high mobility group box 1 (HMGB1) in 5-FU-induced oral mucositis. (**A**) Immunohistochemical analysis of cytosolic HMGB1 expression in the oral mucosa. The number of HMGB1-positive cells per field in the lesion have been count. * *p* < 0.05 vs. 500 mg/kg ANT-rich extract fed with 5-FU injection, # *p* < 0.001 vs. DI fed with normal saline (NS) injection; (**B**) serum HMGB1 levels were evaluated by ELISA. Data are expressed as the mean ± S.D. (*n* = 6). * *p* < 0.001 vs. 1000 mg/kg ANT-rich extract fed with 5-FU injection; (**C**) Correlation of the levels of collagen in the buccal mucosa and serum HMGB1.

5-FU causes leukocyte attraction in the peritoneal cavity by activating autophagy and HMGB1 release [25]. Lipopolysaccharide (LPS) had a synergistic effect with 5-FU, inducing HMGB1 secretion [13]. Additionally, the translocation of nuclear HMGB1 to the cytoplasm during an inflammatory process was distinctly observed in in vivo rat models [26]. In our study, we demonstrated the expression of HMGB1 in the cytosol of keratinized oral mucosa in 5-FU-induced mucositis specimens (data not shown). The quantification of cytoplasmic HMGB1 protein localization showed that the number of HMGB1-positive cells in group B was significantly higher than that of group A (43.83 ± 2.5 and 25.67 ± 2.0; *p* < 0.001) (Figure 6A). Pretreatment with ANT-rich extract for 1000 mg/kg (group D) significantly reduced the cytosol HMGB1 levels in the epithelial cells (21 ± 1.8) compared with group B (*p* < 0.001). Additionally, serum HMGB1 levels were examined in all rats (Figure 6B). The levels of HMGB1 were significantly higher in group B (35.7 ± 9.9) and group C (41.18 ± 10.9) compared to group D (3.8 ± 2.3) (*p* < 0.001). Our results demonstrated that activated cytoplasmic HMGB1 may be released from buccal mucosal tissue into blood circulation. Moreover, a significant negative correlation between the HMGB1 serum levels in group B and the density of collagen in group D (*r* = −0.792, *p* = 0.001) was demonstrated (Figure 6C).

### 2.7. ANT Protect 5-FU-induced Oral Keratinocyte Cell Growth Suppression

**Figure 7:**
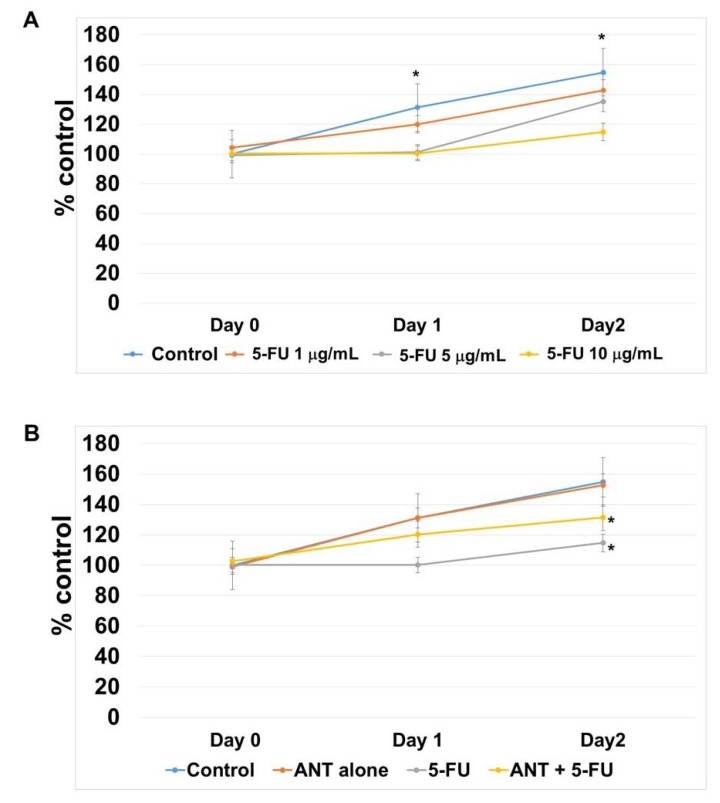
The effect of ANT-rich extract on 5-FU-induced cell growth suppression. Viable cells were estimated by 3-[4,5-dimethylthiazol-2-yl]-2,5 diphenyl tetrazolium bromide (MTT) assay and expressed as percentage of control cells. (**A**) Cells were grown in medium supplemented with 5-FU (1, 5 and 10 μg/mL) for 2 days. 5-FU did not exert cytotoxic effects on oral keratinocytes. Data are expressed as the mean ± SD. * *p* < 0.05 vs. 5 and 10 μg/mL of 5-FU treatment; (**B**) cells were pretreated with 1 mg/mL ANT-rich extract and exposed to 5-FU (10 g/mL) for 2 days. * *p* < 0.05 vs. control.

Based on the results above, we sought to examine the effect of ANT-rich extract on 5-FU in oral keratinocytes in vitro. At first, cell proliferation was determined by 3-[4,5-dimethylthiazol-2-yl]-2,5 diphenyl tetrazolium bromide (MTT) assay. The dose of ANT-rich extract up to 2 mg/mL did not affect the survival or growth rate of the cells (data not shown). The growth inhibitory response of oral keratinocytes to 5-FU was investigated for 2 days (Figure 7A). 5-FU at the concentrations of 5 and 10 μg/mL induced an apparent statistically significant cell growth inhibition (*p* < 0.05) but did not exert a cytotoxic effect. Based on this finding, we chose a 5-FU concentration of 10 μg/mL for use in further studies regarding whether or not ANT-rich extract can restore the suppressive effect of 5-FU on cell growth. As can be seen in Figure 7B, ANT-rich extract alone did not affect cell growth when compared to the growth of the control cells. In addition, the dose of ANT-rich extract (1 mg/mL), which did not affect cell growth when used alone, resulted in an enhanced recovery of cell growth when used in combination with 5-FU. Our findings suggested a protective effect of ANT-rich extract on the amelioration of 5-FU-induced growth suppression.

### 2.8. ANT Suppresses NF-κB p50 and p65 Levels in Nuclear Fraction of Oral Keratinocytes

**Figure 8:**
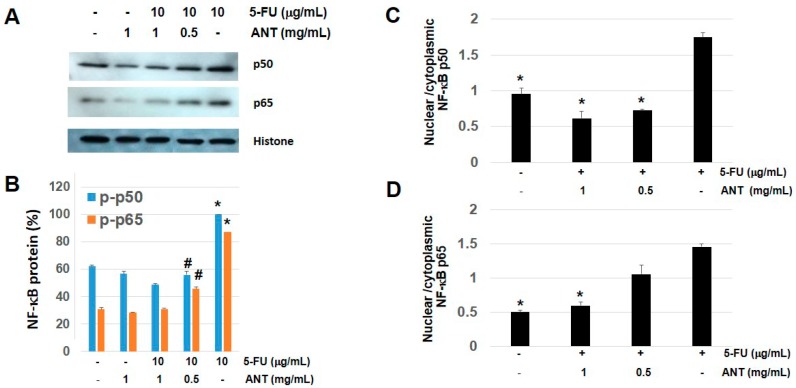
Effects of ANT-rich extract on 5-FU-induced NF-κB phosphorylation. (**A**) Oral keratinocytes were stimulated with 5-FU in the presence or absence of ANT-rich extract for 1 h. Western blotting was performed for nuclear fraction of NF-κB p50 and p65, with histone as an equal loading control. The blots shown are representative of data from at least three experiments; (**B**) Quantifying nuclear NF-κB p 50 and p65 levels; data are expressed as the mean ± S.D. * *p* < 0.01 vs. control, # *p* < 0.05 vs. 1 mg/mL ANT-rich extract in the presence of 5-FU; the nuclear/cytoplasmic intensity ratio of (**C**) NF-κB p50 and (**D**) NF-κB p65. ANT-rich extract abundantly abrogated NF-κB nuclear translocation in response to 5-FU. Data are expressed as the mean ± S.D. * *p* < 0.05 compared to the 5-FU-treated cells.

Our study demonstrated that NF-κB activation was found in the biopsy of rat oral mucosae treated with 5-FU; we therefore evaluated whether ANT-rich extract inhibited 5-FU-induced NF-κB p50 and p65 activation. Oral keratinocytes were treated with 0.5 and 1 mg/mL ANT-rich extract and/or 10 g/mL 5-FU for 1 h, and isolated nuclei were analyzed for the kinetics of NF-κB activation (nuclear translocation), assessed by Western blotting (Figure 8A). 5-FU significantly increased the nuclear translocation of NF-κB p50 and NF-κB p65 as compared to the control (*p* < 0.01). Following 0.5 mg/mL of ANT-rich extract treatment, we observed 44.2% and 41.4% inhibition of NF-κB p50 and p65 phosphorylation, respectively (Figure 8B). Moreover, in the cells treated with 1 mg/mL ANT-rich extract, the inhibition values of NF-κB p50 (51.3%) and p65 (56.0%) were significantly higher (*p* < 0.05) than cells treated with 0.5 mg/mL ANT. There was no statistically significant difference in NF-κB p50 and p65 levels between untreated cells and ANT-rich extract alone-treated cells (*p* > 0.05). The expected decrease in nuclear/cytoplasmic ratio was also evident across the ANT-rich extract dose range, while ANT-rich extract abundantly abrogated NF-κB p50 (Figure 8C) and NF-κB p65 (Figure 8D) nuclear translocation in response to 5-FU.There was a statistically significant difference (*p* < 0.05) present between 0.5 and 1 mg/mL of ANT-rich extract treatment regarding the nuclear/cytoplasmic ratio of NF-κB p50 levels, whereas treatment of cells with 0.5 mg/mL ANT-rich extract did not affect the nuclear/cytoplasmic ratio of NF-κB p65. These data are in agreement with our results in the animal model (Figure 5) indicated that 5-FU increased the phosphorylation of NF-κB p50 and p65 and is thereby suppressed by ANT-rich extract treatment.

## 3. Discussion

Studies on mucositis using animal models focus on continuing the search for new therapies for oral mucositis. The results from this study suggest that ANT-rich extract demonstrates significant anti-inflammatory properties and oral mucosa wound healing through the inhibition of NF-κB p50 and p65 signaling in 5-FU induced oral mucositis rat model and in the oral keratinocyte culture. To the best of our knowledge, this study demonstrates for the first time that ANT-rich extract from black rice (*Oryza sativa* L.) is able to protect rats from post-chemotherapy oral mucositis and suppress the cell growth inhibitory effect of 5-FU. The results demonstrate a protective effect of ANT-rich extract on 5-FU induced oral mucositis, which was consistent with previous studies stating that ANT significantly lowered chemotherapy-induced intestinal mucositis [20] and that ANT-rich extract from bilberry exhibited a protective effect against the myelotoxicity caused by 5-FU [27].

Microorganisms inhabiting the oral cavity can enter the system through the ulcerative lesions seen in oral mucositis, consequently causing systemic infection in cancer patients receiving chemotherapy [28]. Additionally, our findings revealed a significant loss of body weight in rats treated with 5-FU as early as day 14, suggesting a systemic repercussion of experimental oral mucositis, consistent with previous studies in a hamster model [29]. However, gavage feeding with ANT did not cause any alterations in the average body weights of rats. In the present study, the chromatogram of purified products following ANT detection showed that C3G was the predominant ANT present in Thai *Oryza sativa* L. The minor ANT detection was P3G. The ANT composition identified in our black rice extract was consistent with previous reporting [30]. In our study, ANT was extracted in ethanol and contained C3G ≈ 0.85 mg/g and P3G ≈ 0.076 mg/g of the dry extract. The FRAP value and total phenolic content of the ANT-rich extract is 27.23 mM Fe (II) equivalents/gFW and 181.73 mM GAE/gFW, respectively. However, Sompong, et al. demonstrated tthe C3G (5.69 mg/g) and peonidin3-glucoside (11.46 mg/g) contents in black rice using HCl solvent in methanol (85:15, (*v*/*v*) [31]. Their total phenolic contents values (300–600 mM GAE/gFW) were also higher than those of our extracts. The previous study on the ANT content of different rice varieties demonstrated that Thai red rice, Niaw Dam Pleuak Khao and Niaw Dam Pleuak Dam, followed by China black rice, has a superior quality with respect to its ANT compositions [31]. The higher amount of ANT content and antioxidant capacities from the previous study compared to our findings are probably due to differences in rice cultivars, as well as varying methods of extraction, separation, purification, and analysis between the studies.

Previous study showed that rats receiving ANT-rich extract from *Oryza sativa* L. Japonica at 500 mg/kg had an effect against liver damage by alcohol [21]. Before beginning the current experiments, different concentrations (250, 500, 750, and 1000 mg/kg) of the same ANT extracts were tested. C3G is considered as a powerful antioxidant with pharmacological benefits including antioxidant properties [21] and anti-inflammatory effects [17,18,19]. We report for the first time that the highest ANT dose (1000 mg/kg body weight) from Thai black rice, which was gavage-fed once daily, contains approximately 0.85 mg/kg; C3G shows maximum protective and healing effects on oral mucositis induced by 5-FU. Apart from total phenolic and anthocyanin content, black rice contains high levels of flavonoids; more phytochemical analysis and the pharmaceutical activity of this plant should be further investigated.

5-FU is a common chemotherapeutic drug that causes oral mucositis [4]. 5-FU injected rats have been demonstrated to be at a high risk of oral mucositis [5,32]. Recent studies have pinpointed NF-κB as having a key role in the pathogenesis of mucositis [3,33,34]. In our study, we detected an abundance of activated NF-κB p50 and p65 in nuclear-stained oral keratinocytes obtained from rats injected with 5-FU. The exact mechanism(s) by which 5-FU causes oral mucositis are still unknown; however, 5-FU has been reported as an inducer of NF-κB [24]. Therefore, the abundant amount of activated NF-κB p50 and p65 seen in oral keratinocytes and inflammatory cells in the granulation tissues of rats treated with 5-FU alone suggests the importance of NF-κB in the pathogenesis of 5-FU-induced oral mucositis. In agreement with our investigation, previous studies have shown strong associations between NF-κB-mediated biological manipulation of the oral mucosa and mucositis progression [35].

In the present investigation, we demonstrated that ANT-rich extract significantly reduced the lesions induced by 5-FU in the oral mucosa. The data provided in this study strongly suggest that ANT-rich extract given prior to chemotherapy favorably affects the severity and duration of ulcerative mucositis induced by 5-FU. The macroscopic and microscopic effects were associated with reduced inflammatory infiltration and epithelialization. Accordingly, these histopathologic findings were presented in a previous study [27], although there were variations in the parameters used for biochemical analysis, plant species, and ANT content in the study mentioned. The results herein further verify the anti-inflammatory role of ANT on NF-κB and HMGB1 expression following 5-FU-treatment by immunohistochemical analysis and ELISA. Thus, the protective role of ANT found in the present study may be explained by its capacity to inhibit NF-κB p50 and p65 activation, thereby inhibiting the release of inflammatory cytokines that mediate 5-FU-induced oral mucositis. These findings are consistent with previous observations in animal and clinical studies, where ANT demonstrated anti-inflammatory effects by the inhibition of NF-κB transactivation [17] and decreased plasma concentrations of pro-inflammatory mediators in human subjects [18].

The primary damage of oral mucositis response describes an activation of transcription factors such as NF-κB leading to the production of pro-inflammatory cytokines such as TNF-α, IL-1β, and IL-6, which cause damage to both epithelium and connective tissue [2,11]. Our results demonstrate that the ANT-rich extract partially protects against 5-FU-induced cellular stasis in oral keratinocytes, and restores oral mucosal surfaces in vivo via immunological modulatory effects. Future studies should assess the predictive potential of ANT in determining associated cytokine production in vitro using peripheral blood mononuclear cells.

In the present study, erythema, hyperemia, hemorrhagic areas, and extensive ulcer and abscesses appeared in the cheek pouches of rats which were subjected to 5-FU injection by day 14, and their degree was maximal by day 17. Wound healing involves complex cellular and molecular interactions. There are five phases involved in the pathogenesis of mucositis, including initiation, primary damage and message generation, signal amplification, ulceration, and healing phase [11]. Following the initiation of the healing cascade by direct injury to DNA, a series of transcription factors were prompted to respond to the primary damage, which triggered a consequence of cytokine release. In the final stage of the healing process, collagen deposition and remodeling occurs. Our findings demonstrated a slight decrease in the macroscopic scores of mucositis in rats which were treated with ANT-rich extract (groups C and D) compared to group B on day 17 of the experiment. However, a significant decrease in these scores (*p* < 0.01) was observed on day 29 in rats which were treated with ANT-rich extract for 1000 mg/kg (group D) compared to group B. In addition, ANT-rich extract treatments attenuated NF-κB activation, suppressed the elevation of HMGB1 expression, and induced collagen formation. Thus, from our findings, ANT-rich extract treatments may not result in direct repair during the initiation stage of the wound healing but promote a permeability of collagen to the lesion, epithelial cell proliferation and differentiation, and repair the destructive mucosa during the late stage. Nevertheless, specific growth factors such as nerve growth factor [36] or drugs such as corticosteroids [37] which take part in the effective wound healing in the oral cavity should be employed to compare the efficacy with ANT-rich extract.

The present study is, as far as we know, the first investigation into the protective effects of ANT in 5-FU–induced oral mucositis and its involvement in protecting oral keratinocytes from the growth inhibitory effect of 5-FU. Mucositis can affect the entire mucosal lining of the gastrointestinal tract, with oral and oropharyngeal mucosae being common sites of disease occurrence [38]. Our findings in vitro are consistent with animal studies demonstrating that the nuclear portions of NF-κB p50 and NF-κB p65 were activated by 5-FU, then suppressed following ANT-rich extract administration. However, the molecular mechanisms in other cells involved in oral mucositis such as oral fibroblasts should be further investigated [39]. With these outcomes, further clinical studies on the anticancer activities of black rice extract should be performed.

In conclusion, our results highly suggest that ANT found in Thai *Oryza sativa* L. extracts possess anti-inflammatory and protective effects against experimental models of mucositis induced by 5-FU. We speculate that these anti-inflammatory effects are related to NF-κB suppression. The possibility of using this extract in the treatment of human oral mucositis merits further investigation.

## 4. Materials and Methods

### 4.1. Materials and Chemicals

NF-κB p50 and p65 Abs were purchased from Santa Cruz (Santa Cruz Biotechnology, Inc., Santa Cruz, CA, USA). HMGB1 Ab and cell fractionation kit were obstained from Abcam (Cambridge, MA, USA). Unless otherwise stated, all other reagents were supplied by Sigma-Aldrich Inc. (St. Louis, MO, USA).

### 4.2. Plant Material and Extraction

Black rice was obtained from Chachoengsao Province, Thailand. ANTs in Thai black rice were extracted in ethanol (60/40, *v*/*v*%) and concentrated at 40 °C (extraction yield 3.5% of dry weight) using a Buchi B-490 rotary evaporator (BÜCHI Labortechnik AG, Flawil, Switzerland), lyophilized with a freeze-dryer (Labconco Corp., Kansas City, MO, USA), and the crude extract was stored at 4 °C. Purified extracts were prepared following previous reporting [40]. Briefly, a C18 Sep-Pak cartridge (Waters Corp., Milford, MO, US) was activated with DI and methanol (Merck KGaA, Darmstadt, Germany). The crude ANT extract was then loaded onto the column. Following successive washes, the ANTs were eluted with methanol containing 0.01% HCl. The ANT solution was then collected and condensed at 40 °C using a Büchi B-490 rotary evaporator under vacuum.

For HPLC-electrospray ionization (ESI) mass spectrometry (MS), ANT-rich extracts were separated and quantified by reverse-phase HPLC (Agilent model Model:1100 (Binary pump, Degasser, Autosample, DAD) using a Hypersil Gold C18 column (inner diameter, 3 μm; 4.6 × 150 mm; Thermo Fisher Scientific Inc., Waltham, MA, USA) following previous reporting [40], with slight modifications. The column was eluted with a mobile phase consisting of formic acid (VWR International, Ltd., Leicestershire, UK) and methanol at a flow rate of 1 mL/min. The separated ANTs were detected and measured at 530 nm, and were identified based on their retention times and ultraviolet (UV)-visible (Vis) wavelength spectra of pure authentic standards (Sigma, St. Louis, MO, USA). The identity of each peak was verified. The chromatographic system was coupled to an ion trap mass spectrometer (Esqurire 3000+, Bruker Daltanic Model, Billerica, MA, USA) equipped with an ESI working in positive ion mode. The LC-MS was eluted with formic acid and methanol.

### 4.3. Quantification of ANT by UV-Vis Spectroscopy and Antioxidant Activities

The ANTs were quantified by UV-Vis spectroscopy as previously described [14]. The model reaction solution was diluted with 0.01% HCl in distilled water. Absorbance at 510 nm was compared with that of known standard solutions using a Genesys 10 UV spectrophotometer (Thermo Scientific, Grand Island, NY, USA). The determination of total phenolic content was determined using Folin–Ciocalteu reagent (FRC) as previously described [40], with minor modifications. The absorbance of the mixture was measured at 765 nm using a UV-Vis Genesys 10 UV spectrophotometer. A standard curve was plotted using gallic acid (0.07–10 mg/mL in methanol; Sigma) as a standard. The total phenolic content was expressed as gallic acid equivalents (mM GAE/gFW). Ferric-reducing antioxidant power (FRAP) was measured as per previous reporting [40]. The absorption was measured at 595 nm using a spectrophotometer (Epoch; Biotek, Winooski, VT, USA) with the Gen 5 Data analysis software interface (https://www.biotek.com/). Aqueous or methanol solutions containing known Fe (II) concentrations were used to calibrate the FRAP assay. FRAP values, expressed as mM of Fe(II) equivalents/gFW), were determined by comparing the change in absorption of the test mixture with those of the Fe(II) standards. Total antioxidant activity was determined using the OxiSelect TAC Assay kit (Cell Biolabs, San Diego, CA, USA) following manufacturer’s instructions.

### 4.4. Animal Model

Healthy male Wistar rats (7 weeks, 280–300 g) from the National Laboratory Animal Center, Mahidol University, Nakhon Pathom, Thailand were used in this study. Animals were maintained in an animal room with a standard environmental condition of 22 ± 2 °C, 50 ± 5% relative humidity, a 12 h/12 h light/dark cycle with water and food ad libitum. All animal experiments were performed according to the guidelines for animal care and use of Mahidol University Animal Care and Use Committee. All animal experiments were approved by the Ethical Clearance Institutional Animal Care and Use Committee (Faculty of Pharmacy, Mahidol University, Bangkok, Thailand) in 8 May 2014 (certificate No: PYT011/2557).

### 4.5. Induction of Experimental Oral Mucositis

Oral mucositis was induced following the protocol proposed by Sonis et al [32]. Three intraperitoneal (i.p.) injections of 5-FU 60 mg/kg were administered once a day at a five-day interval (days 7, 12, 17) for 29 days. On day 11, the right cheek pouch of all animals was irritated by superficial scratching with a tip of an 18-gauge needle under isoflurane anesthesia to mimic the clinical effect of chronic irritation. The needle was dragged twice in a linear fashion across the everted right cheek pouch. This technique has been used repeatedly to induce ulcerative mucositis, which is similar to human oral mucositis.

### 4.6. Animals and Study Design

Wistar rats were randomly divided into five groups, as follows: group A: gavage-fed 1 mL deionized water (DI) and injected normal saline (NS) by i.p. injection, serving as a negative control group; group B: gavage-fed 1 mL (DI) and injected with 60 mg/kg 5-FU by i.p. injection, serving as a positive control group; group C: gavage-fed 500 mg/kg ANT-rich extracts once daily and injected with 60 mg/kg 5-FU i.p.; group D: gavage-fed 1000 mg/kg ANT-rich extracts once daily and injected with 60 mg/kg 5-FU i.p.; and group E: gavage-fed 1000 mg/kg ANT-rich extracts once daily and injected with NS i.p. The beginning of the gavage was considered to be day 1 of the study. ANT-rich extracts were fed 1 week before the first dose of 5-FU injection. The animals were observed daily and weighed once every two days. For the purpose of observation, rats were anesthetized with isoflurane and the buccal pouches were everted and photographed. Subsequently, buccal mucosa was biopsied and tissue samples were collected for histochemical analysis and immunohistochemistry for p50 and p65 subunits of NF-κB, and HMGB1. Blood was collected for the assessment of serum HMGB1 level.

### 4.7. Macroscopic Analysis of Buccal Mucosae

On day 29, the animals were anesthetized with isoflurane inhalation, and the buccal pouches were photographed for macroscopic scoring based on a valid macroscopic scoring system described by Lima et al. [41]. For macroscopic analysis, inflammatory aspects such as erythema, hyperemia, hemorrhagic areas, epithelial ulcerations, and abscesses were evaluated by three examiners and graded as follows: score 0: normal cheek pouch with erythema and hyperemia absent or discrete, no hemorrhagic areas, ulcerations, or abscess; score 1: moderate erythema and hyperemia, no hemorrhagic areas, ulceration, or abscess; score 2: severe erythema and hyperemia, presence of hemorrhagic areas, small ulceration, or scarred tissue, but no abscess; and score 3: severe erythema and hyperemia, presence of hemorrhagic areas, extensive ulcerations, and abscess of the buccal mucosa.

### 4.8. Histopathological Analysis

In each experimental group, the samples of buccal pouches were removed by incisional biopsy for histopathological analysis. The specimens were fixed in 10% neutral buffered formalin, dehydrated, and embedded in paraffin. Sections of 6 μm thickness were obtained. Mallory’s azan staining was used to visualize collagen fibers, and Mayer’s hematoxylin stain was used to examine the parameters of inflammatory cell infiltration, vasodilation, presence of hemorrhagic areas, edema, ulcerations, and abscesses [42]. Histopathological sections were analyzed under light microscopy. Density of collagen bundles and epithelial thickness were quantified using Image J software (ImageJ, US National Institutes of Health, Bethesda, MD, USA) from six randomly selected areas of the same section under high power fields (×400). The average density of collagen was calculated as Integrated Density (the product of Area and Mean Gray Value)/Area [43]. Calculations of the average epithelial thickness in the Mayer’s hematoxylin staining samples were based on 20 measurements for each time point.

### 4.9. Immunohistochemistry for p50 and p65 NF-κB and HMGB1

Sections were deparaffinized by xylene and rehydrated. Immunohistochemistry was performed as described previously for NF-κB p50, p65 and HMGB1 [20]. Sections were incubated with their respective primary Abs diluted in PBS containing 0.1% bovine serum albumin overnight at 4 °C. Monocolonal NF-κB p50, p65 and HMGB1 Abs were used at a dilution of 1:500. Following washing with PBS, the sections were incubated with corresponding HRP-conjugated secondary Abs for 1 h at RT, followed by staining with diaminobenzidine (DAB) (Histofine MAX-PO kit, Nicherei, Tokyo, Japan). Control staining was performed with non-immune IgGs and slides stained with DAB (DakoCytomation; Glostrup, Denmark) and counterstained for 2 min with hematoxylin (DakoCytomation). Sample was visualized by light microscopy (LEICA Microsystem GmbH, Wetzlar, Germany). A total of four digital images per group/time point under high-power fields (×400) were assessed by a calibrated examiner blinded to the protocol. A modified semi-quantitative method was used to count the NF-κB p50, p65 and HMGB1 positive cells as previously described [12].

### 4.10. Cell Culture Conditions

Oral keratinocytes were obtained from ScienCell Research Laboratories (San Diego, CA, USA). Cells were maintained in complete oral keratinocyte medium supplemented with keratinocyte growth supplement and penicillin/streptomycin solution. Cells were maintained at 37 °C in a humidified atmosphere containing 5% CO_2_ and 95% air. All cells were cultured in serum-free Opti-MEM-I medium (Gibco, Grand Island, NY, USA) for at least 15 h before treatment to eliminate the possible side effect of growth factors.

### 4.11. Cell Viability Test

Cell viability was performed by MTT (3-(4,5-dimethylthiazol-2-yl)-2,5-diphenyltetrazolium bromide) assay according to a method previously described [42]. Briefly, cells were treated with ANT-rich extracts for 1 mg/mL and 5-FU for 1, 5, and 10 μg/mL for 2 days and incubated with MTT (0.5 mg/mL) for 3 h. Formazan crystal was solubilized by adding DMSO for 16 h.

### 4.12. Preparation of Nucleic/Cytosolic Fractions

Cells (2 × 10^5^ cells) were cultured in 60 mm dish and were pretreated with ANT-rich extracts (0.5 and 1 mg/mL) and exposed to 10 g/mL of 5-FU for 1 h. Whole cell lysates were extracted using cell lysis buffer (Cell Signaling Technology, Inc., Danvers, MA, USA) or nucleic/cytosolic fractions were extracted using cell fractionation kit according to the manufacturer’s instructions and stored at −20 °C until use. Protein concentrations were determined by Bradford protein assay using bovine serum albumin as standard (Bio-Rad, Hercules, CA, USA).

### 4.13. Western Blot Analysis

Western blot analyses were carried out as described previously [44]. Briefly, samples were mixed with 2× electrophoresis sample buffer solution with bromophenol blue (Santa Cruz Biotechnology) before being subjected to 12% sodium dodecyl sulfate -polyacrylamide gel electrophoresis (PAGE) and transferred onto nitrocellulose membranes (Schleicher & Schuell, Dassel, Germany). To prevent nonspecific binding, the membrane was blocked with a solution containing 5% (*w*/*v*) nonfat dry milk with 1% (*v*/*v*) Tween 20 in PBS for 1 h at RT. Anti-NF-κB p50 or p65 Ab was incubated for 1 h at RT. The membranes were washed and incubated with horseradish peroxidase-conjugated anti-mouse monoclonal IgG (MP Biomedicals Inc., Solon, OH, USA). Labeled bands were visualized using an enhanced chemiluminescence system (GE Healthcare Bio-Science, Pittsburgh, PA, USA) and exposed to high-performance chemiluminescence film (GE Healthcare). The intensity of the protein bands in Western blotting was quantified using National Institutes of Health Image version 1.63 software (National Institutes of Health, Bethesda, MO, USA).

### 4.14. HMGB1 Measurement by Enzyme-Linked Immunosorbent Assay

HMGB1 levels were quantified using a commercial ELISA kit (Shino-test, Sagamihara, Kanagawa, Japan), according to the manufacturer’s instruction.

### 4.15. Statistical Analysis

Statistical significances between different groups were determined by one-way analysis of variance (ANOVA) test or Student’s paired *t*-test. The correlation among three examiners and the macroscopic scores were calculated using Pearson’s correlation coefficient. *p* values < 0.05 were considered statistically significant. All calculations were performed using SPSS software (version 20.0; SPSS Inc., Chicago, IL, USA).

## Figures and Tables

**Table 1:** The parameters used for ANT peak identification.

Compound No. *	RT (min)	MS, M+ (*m*/*z*)	MS/MS (*m*/*z*)	Assignment **
1	31.0	449.1	287	Cyanidin-3-glucoside
2	37.0	463.1	301	Pelargonidin-3-glucoside

* Diode array detection at 530 nm; ** Based on the fragmentation pattern and its aglycone. The assay was performed in triplicate. RT, retention time; MS, mass spectrometry; M+, molecular ion.

**Table 2:** Antioxidant activities of the ANT in *Oryza sativa* L.

Parameter	Crude Extract	Purified Extract
Total antioxidant capacity (mM/g)	1.07 ± 0.06	1.660 ± 0.297 *
FRAP (mM Fe (II) equivalents/gram of fresh weight)	27.23 ± 1.00	28.06 ± 3.66
Total phenol content (mM Gallic acid equivalent/gFW)	181.73 ± 12.79	193.4 ± 6.71 *

Total antioxidant activity was determined using the OxiSelect Total Antioxidant Capacity (TAC) Assay kit. The total phenolic content was determined using the Folin Ciocalteu reagent, with gallic acid as the standard. FRAP, ferric reducing ability of plasma. The assays were carried out in triplicate and the mean values were recorded. * *p* < 0.05 vs. the crude extract.

## Supplementary Materials

Supplementary materials can be found at http://www.mdpi.com/1422-0067/19/10/2981/s1.
