# Supplementary material for: Anthocyanins Extracted from Oryza sativa L. Prevent Fluorouracil-Induced Nuclear Factor-κB Activation in Oral Mucositis: In Vitro and In Vivo Studies
Source: Int J Mol Sci. 2018 Sep 29;19(10):2981. doi: 10.3390/ijms19102981 (PMC6213925; doi:10.3390/ijms19102981)
Supplement: Supplementary file 1 [file ijms-19-02981-s001.pdf]

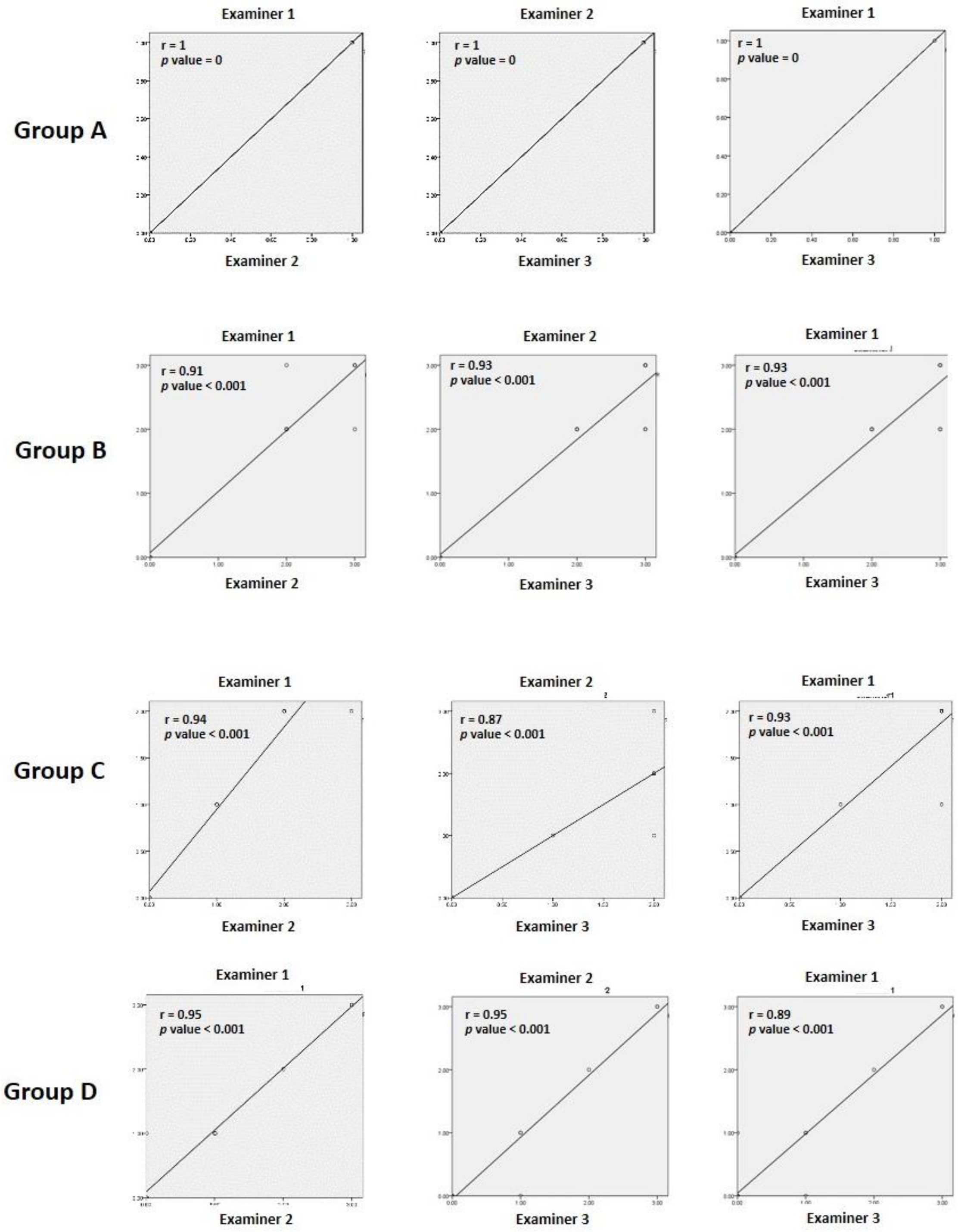

Group A
Examiner 1
r = 1
p value = 0
Examiner 2
Examiner 2
r = 1
p value = 0
Examiner 3
Examiner 1
r = 1
p value = 0
Examiner 3
Group B
Examiner 1
r = 0.91
p value < 0.001
Examiner 2
Examiner 2
r = 0.93
p value < 0.001
Examiner 3
Examiner 1
r = 0.93
p value < 0.001
Examiner 3
Group C
Examiner 1
r = 0.94
p value < 0.001
Examiner 2
Examiner 2
r = 0.87
p value < 0.001
Examiner 3
Examiner 1
r = 0.93
p value < 0.001
Examiner 3
Group D
Examiner 1
r = 0.95
p value < 0.001
Examiner 2
Examiner 2
r = 0.95
p value < 0.001
Examiner 3
Examiner 1
r = 0.89
p value < 0.001
Examiner 3

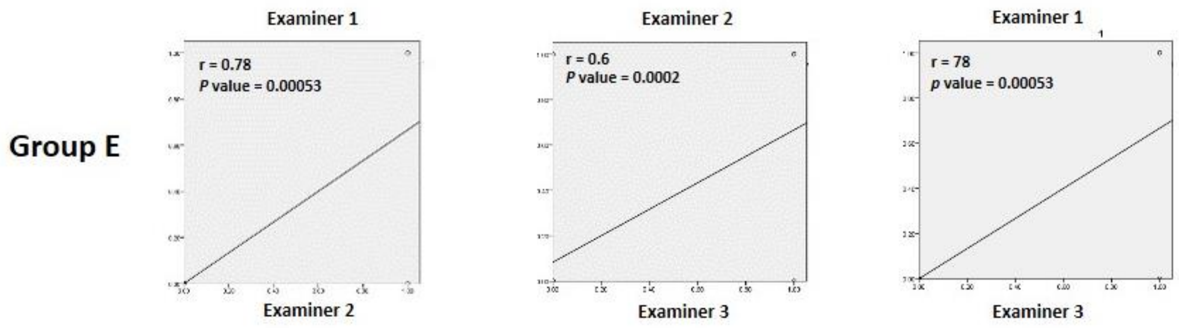

Figure S1: Inter-examiner reliability of macroscopic score measurement. The macroscopic scores on Days 17 and 29 were measured in all groups of rats (A to E) by three examiners. Pearson's correlation coefficient (r) was used to show the correlation between the examiners and *p* values are shown. *p* value < 0.05 was considered statistically significant.

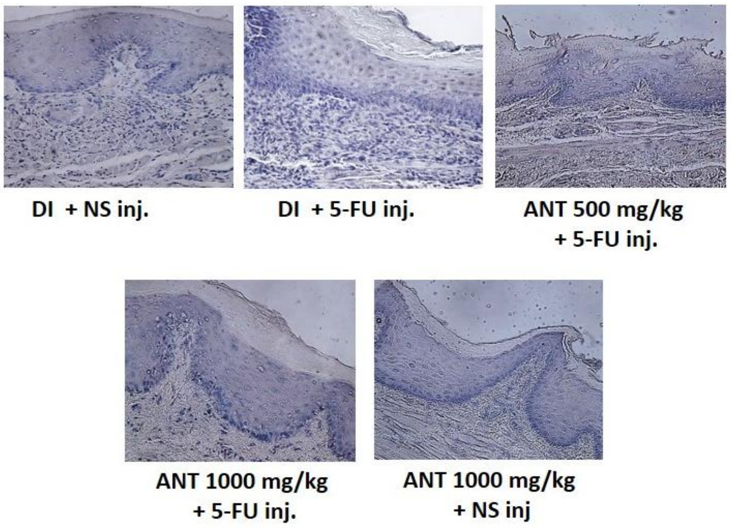

Figure S2: Immunohistochemistry of control isotype- IgG in buccal mucosa after 29 days, oral mucositis was induced by i.p. injection of 5-FU 60 mg/kg. Animals were gavage-fed with ANT 500 mg/kg, 1000 mg/kg or normal saline (NS) daily. Tissues were immunostained with anti-mouse IgG. No positive staining of the tissues in the samples. DI, distriled water. All images were obtained at ×200 magnification.
